# Improved JPEG Coding by Filtering 8 × 8 DCT Blocks

**DOI:** 10.3390/jimaging7070117

**Published:** 2021-07-15

**Authors:** Yasir Iqbal, Oh-Jin Kwon

**Affiliations:** Department of Electrical Engineering, Sejong University, 209 Neungdong-ro, Gwangjin-gu, Seoul 05006, Korea; yasir@sju.ac.kr

**Keywords:** JPEG image coding, image compression, JPEG entropy coding

## Abstract

The JPEG format, consisting of a set of image compression techniques, is one of the most commonly used image coding standards for both lossy and lossless image encoding. In this format, various techniques are used to improve image transmission and storage. In the final step of lossy image coding, JPEG uses either arithmetic or Huffman entropy coding modes to further compress data processed by lossy compression. Both modes encode all the 8 × 8 DCT blocks without filtering empty ones. An end-of-block marker is coded for empty blocks, and these empty blocks cause an unnecessary increase in file size when they are stored with the rest of the data. In this paper, we propose a modified version of the JPEG entropy coding. In the proposed version, instead of storing an end-of-block code for empty blocks with the rest of the data, we store their location in a separate buffer and then compress the buffer with an efficient lossless method to achieve a higher compression ratio. The size of the additional buffer, which keeps the information of location for the empty and non-empty blocks, was considered during the calculation of bits per pixel for the test images. In image compression, peak signal-to-noise ratio versus bits per pixel has been a major measure for evaluating the coding performance. Experimental results indicate that the proposed modified algorithm achieves lower bits per pixel while retaining quality.

## 1. Introduction

In parallel with developments in the field of image capture technologies, data storage is becoming a significant issue encountered by computer and mobile users. Many encoding methods for image data storage have been developed, which can be divided into lossy and lossless methods. Approaches using various techniques to compress data for storage without losing any bits of information in the original image (often captured by a camera or sensor) are called lossless image encoding methods. Examples [1] of lossless methods include GIF (graphics interchange format), JBIG, and PNG (portable network graphics). In contrast, methods using various techniques to store data such that some unimportant details are lost while retaining visual clarity on users’ displays are called lossy image coding methods. Examples of lossy methods include JPEG and BPG (better portable graphics).

Each method has its own advantages and disadvantages. In the field of image compression, methods are evaluated by complexity, compression ratio, and quality of the image obtained. Every method aims to obtain a higher compression ratio, higher quality, and less complexity. Over the previous decade, many methods have competed to obtain a better result. Concerning the above factors and associated trade-offs, JPEG has consistently been the leading image coding standard for lossy compression up to the present day.

Many methods currently provide much better results, but JPEG has been used for the past two decades and still dominates the market. For example, it has been claimed that BPG is likely to overcome JPEG, but this does not seem possible soon. BPG is more complex [2] and thus takes a much longer time to decompile. It was created using high-efficiency video coding (HEVC), which is patented by a company called MPEG LA. It is commonly expected to require considerable time for BPG to be popularly integrated into existing and future computer systems on the market.

In this study, we propose a method to increase the compression ratio of JPEG images without affecting their quality.

## 2. Related Work

### 2.1. JPEG Image Coding Standard

**Figure 1:**
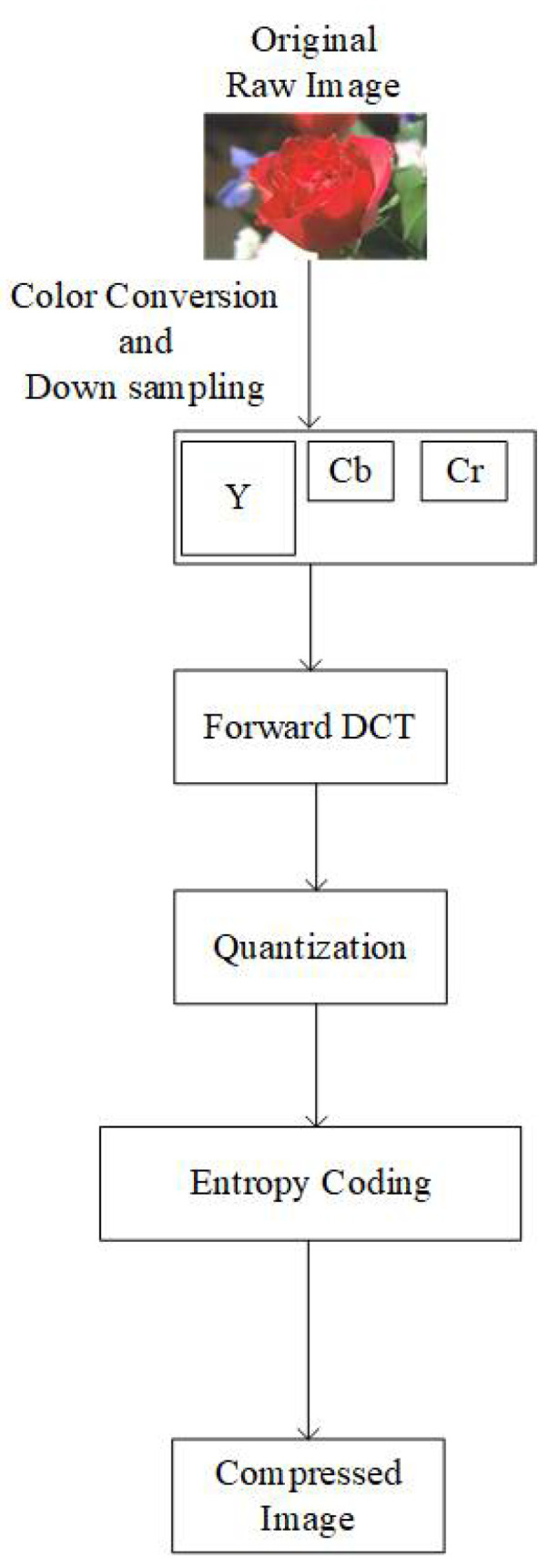
Overview of the conventional JPEG encoder.

The JPEG standard was created in 1992. For a detailed study, readers are referred to [3,4]. Figure 1 shows the basic overview of the conventional JPEG encoder. YCbCr color components are obtained from the raw input image in the first step. Based on user choice, chroma components Cb and Cr are downsampled to the 4:2:2 or 4:2:0 type [4]. Each channel is divided into 8 × 8 blocks. A discrete cosine transform (DCT) is applied on the 8 × 8 blocks in the order from left-to-right and top-to-bottom.

After the DCT, blocks are forwarded for quantization. Luma and Chroma components are quantized using different quantization tables [3]. Quantization tables are generated based on quality factors (QF). The compression ratio and quality of the image are controlled by the QF value. To reduce the redundancy of consecutively occurring DC coefficients, the differential pulse code modulation (DPCM) method is used. In the end, all the processed data is forwarded to the entropy coding module.

### 2.2. Entropy Coding

Data obtained after quantization need to be stored without losing any information. However, instead of saving the data as-is, JPEG compression performs an additional step of entropy coding. Entropy coding achieves additional compression by encoding the quantized DCT coefficients more efficiently based on their statistical characteristics [1]. An individual JPEG compression process uses one of two available entropy coding algorithms, either Huffman [5] or arithmetic encoding [6].

#### 2.2.1. Huffman

Huffman coding is an entropy encoding algorithm using a variable-length code table. This table has been derived based on the estimated probability of occurrence for each possible value of the source symbol (such as a character in a file) [1]. The principle of Huffman coding is to assign lower bits to the more frequently occurring data [7]. A dictionary associating each data symbol with a codeword has the property that no codeword in the dictionary is a prefix of any other codeword in the dictionary [8].

In the JPEG encoder, Huffman coding is combined with run-length coding (RLC) and is called the run-amplitude Huffman code [9]. This code represents the run-length of zeros before a nonzero coefficient and the size of that coefficient. The code is then followed by additional bits precisely defining the coefficient amplitude and sign [4,9]. The end-of-block (EOB) marker is coded when the last nonzero coefficient occurs. This strategy is omitted in the rare case that the last element of the 8×8 block is nonzero. In the case of an empty block, i.e., where all AC coefficients are zero, the encoder codes an EOB.

#### 2.2.2. Arithmetic

Compared to Huffman coding, arithmetic coding bypasses the mechanism of assigning a specific code to an input symbol. An interval (0, 1) is divided into several sub-intervals based on the occurrence probability of the corresponding symbol. The ordering sequence is known to both the encoder and decoder. In arithmetic coding, unlike Huffman coding, the number of bits assigned to encode each symbol varies according to their assigned probability [10]. Symbols with lower probability are assigned higher-bit encodings compared to symbols with higher probability, and their probability decreases in inverse proportion to the probability of the occurrence of the character [1]. The key idea of arithmetic encoding is to assign each symbol an interval. Further, each symbol is divided into subintervals equal to their probability [11].

Both Huffman and arithmetic encoding are performed on the data without filtering out empty AC coefficient blocks, which decreases the compression ratio.

## 3. Proposed Algorithm

**Figure 2:**
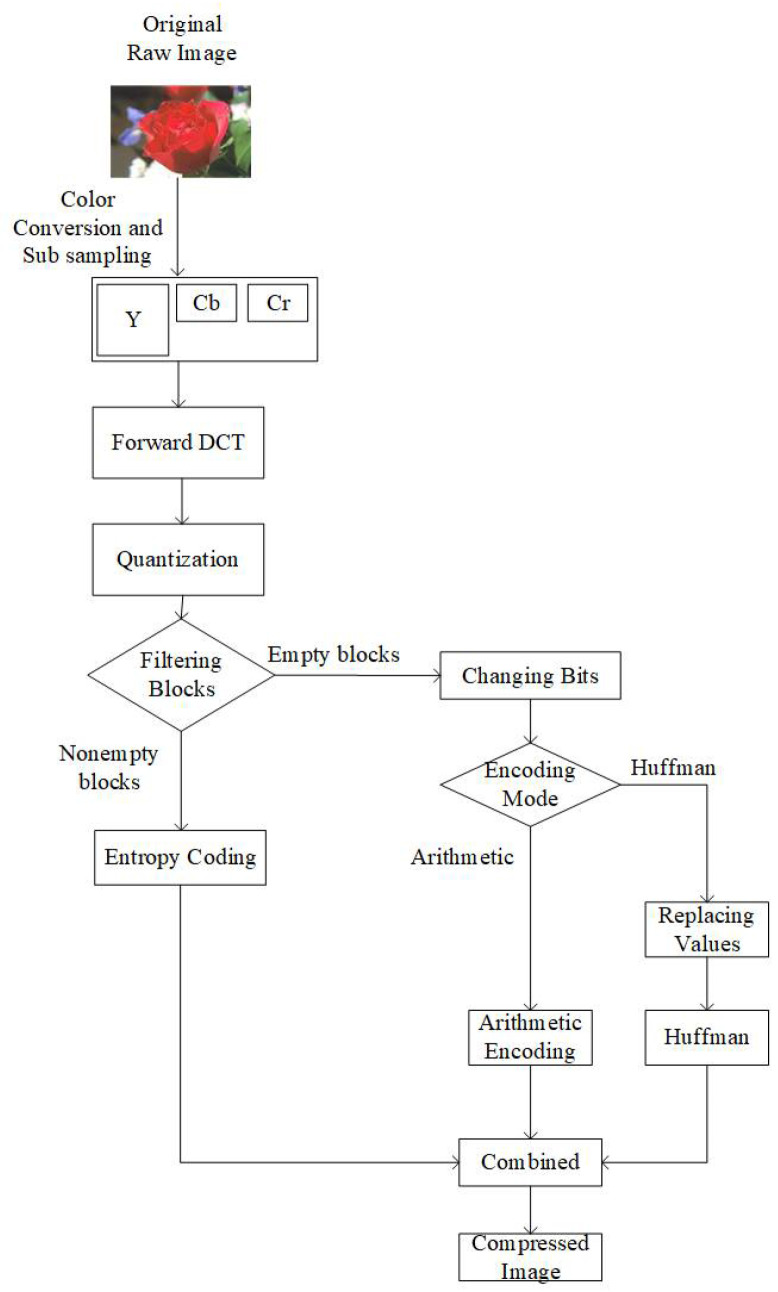
Overview of the proposed JPEG encoder.

Our proposed algorithm is based on the filtration of 8 × 8 blocks. Figure 2 shows an overview of the proposed JPEG image coding. To maintain equivalent complexity between the conventional and our proposed entropy coding, we used separate modes for arithmetic and Huffman coding. Before forwarding the 8 × 8 blocks to the JPEG entropy encoder, we perform three steps. These three steps are named as (1) filtration of blocks, (2) changing bits, and (3) replacing values. The third step (Section 3.3) is performed only in the case of the Huffman encoding mode. It should be noted that the whole process explained in this section is lossless. During the decoding process, we perform the inverse of these steps, and at the end of the inverse process, we know about the location of empty blocks. Moreover, this process has no additional consequences, as we do not change any of the coefficient values.

### 3.1. Filtration of Blocks

In our proposed algorithm, instead of allowing the encoder to encode the EOB marker for the empty blocks along with the array of non-empty blocks, all the empty blocks are filtered out, and information on the location of empty and non-empty blocks is stored in a separate binary buffer. In this buffer, we store 0 for empty blocks and 1 for non-empty blocks. In the JPEG encoder, the Y component is compressed using a different quantization table compared to the Cb and Cr components. Due to the different nature of their compression, we use separate buffers for the Y, Cb, and Cr components at this stage. Finally, all buffers for Y, Cb, and Cr are concatenated.

### 3.2. Changing Bits

After concatenating Y, Cb, and Cr buffers, we improve the consistency of identical bit sequence occurrences by replacing all the bit values with 0, except the initial bit 1, only in the case where the next bit is different from the current. In this process, identical occurrences of either 0- or 1-bit values are saved as 0. Thus, we further increase the occurrence of zero bits. For example, suppose we have a sequence of bits as “000011110111”. We have four consecutive zeros followed by four consecutive ones, indicating that a change occurs at the 5th bit in the sequence. Then, we can observe that the next change occurs at the 9th and 10th bit in the sequence. Hence, the sequence is transformed to “000010001100” with bit 1 placed where the change in the sequence occurs.

In the case of arithmetic encoding mode, after performing this step, we provide our buffer to the binary arithmetic encoder [12]. The remaining of the 8 × 8 blocks, where a nonzero AC coefficient existed, were encoded in a conventional way. After the compression process was completed, we appended our compressed buffer to the remainder of the encoded file. 

### 3.3. Replacing Values

**Figure 3:**
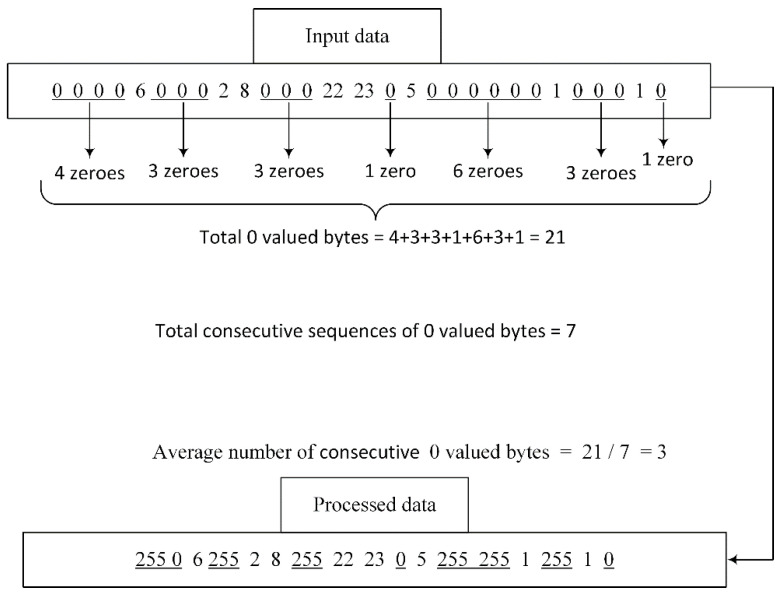
Replacement of values.

This step is performed only when the selected encoding mode is Huffman. By observing the nature of Huffman encoding, we can save more space if we convert our data, resulting from Section 3.2, from bits to bytes. Thus, before replacing the values, bits are converted into bytes. After the conversion, there are still long sequences of consecutive zero-valued bytes present, and to get rid of those long sequences, we perform the step of replacing values. Firstly, we calculate the average number of consecutive zero-valued bytes. The average is used because different types of images have different data. For example, in the case of homogeneous images, the average number of consecutive zeros should be higher owing to a larger amount of empty 8 × 8 blocks present consecutively, whereas, in the case of more detailed images, a smaller number of consecutive empty 8 × 8 blocks are present. The number of consecutively occurring zero-valued bytes equal to the calculated average number is replaced with a less frequently occurring byte value, i.e., 255. In the example shown in Figure 3, there are two data buffers; input data is the data obtained after converting the values from bits to bytes, while the other one is the processed data. 

The total number of zeros was equal to 21 in the original data buffer. These 21 zeros occurred in seven sequences of consecutive zeros. To obtain the average number of consecutively occurring zeros, we divided the total number of zeros with the number of consecutively occurring sequences. Thus, we obtained an average number of zero-valued bytes of three and replaced all three consecutively occurring sequences of three zeros with a constant value of 255.

In the case of floating-point results after division, we round it to the nearest integer. If a byte value of 255 occurs in the input data, we tail it with an additional byte value, e.g., 217, in order to differentiate between a replaced value of 255 and an input data value of 255. The example in Figure 3 demonstrates that after replacement, a sequence with a data size of 29 bytes was reduced to 19 bytes.

As the third step is performed only in the case of JPEG Huffman mode, the processed data is designated for Huffman encoding. After the compression process was completed, we appended our compressed buffer to the remainder of the encoded file.

## 4. Experimental Results

**Figure 4:**
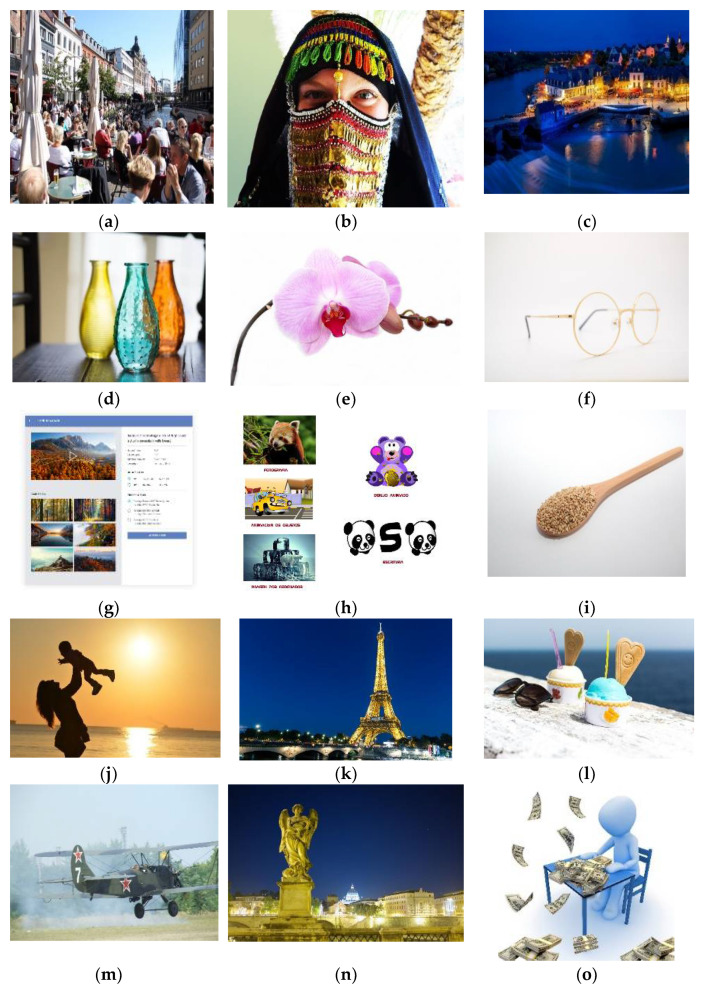
Test images used in experiment: (**a**) Street View, (**b**) Woman, (**c**) Night Scene (**d**) Jars, (**e**) Flowers, (**f**) Glasses, (**g**) Screenshot1, (**h**) Screenshot2, (**i**) Spoon, (**j**) Sunset, (**k**) Paris, (**l**) Ice Cream, (**m**) Air Jet, (**n**) Statue, (**o**) Icon.

**Figure 5:**
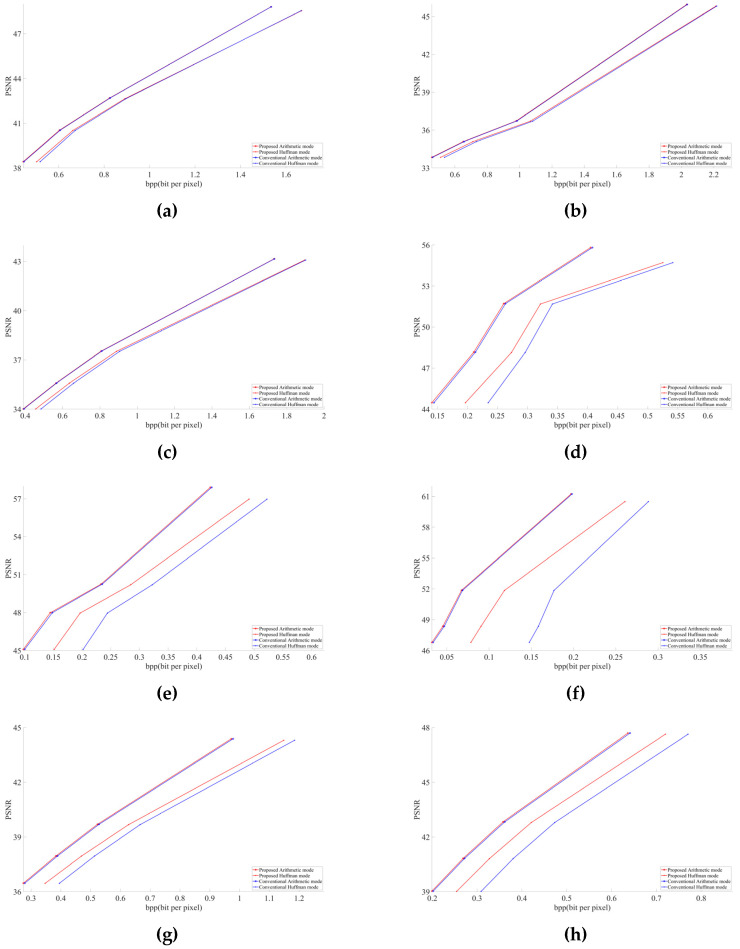

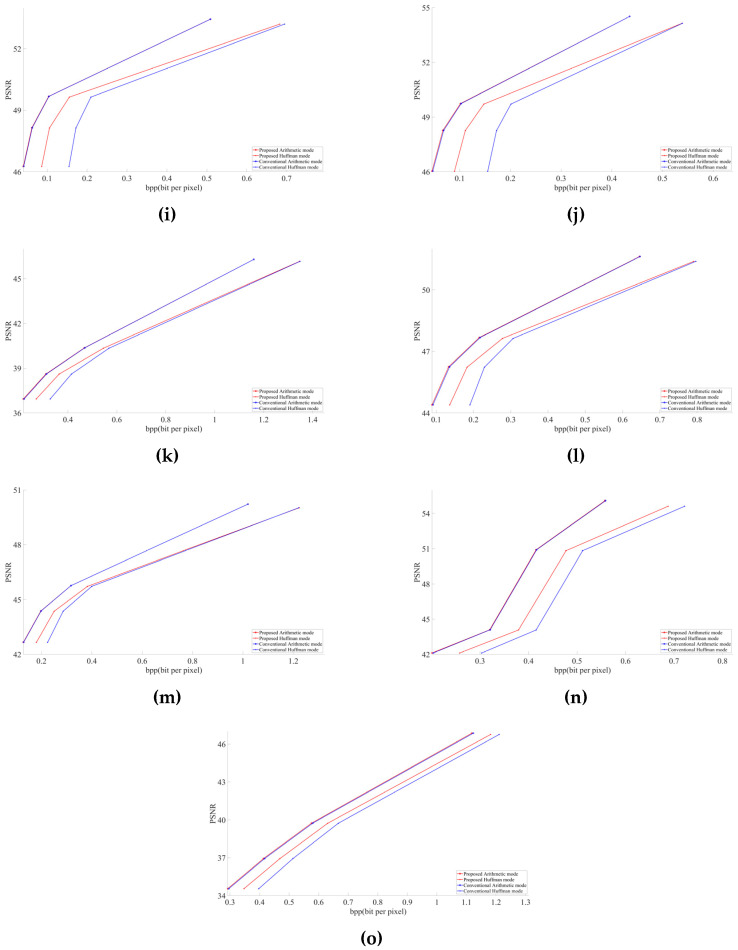
PSNR results of test images shown in Figure 4: (**a**) Street View, (**b**) Woman, (**c**) Night Scene (**d**) Jars, (**e**) Flowers, (**f**) Glasses, (**g**) Screenshot1, (**h**) Screenshot2, (**i**) Spoon, (**j**) Sunset, (**k**) Paris, (**l**) Ice Cream, (**m**) Air Jet, (**n**) Statue, (**o**) Icon.

**Figure 6:**
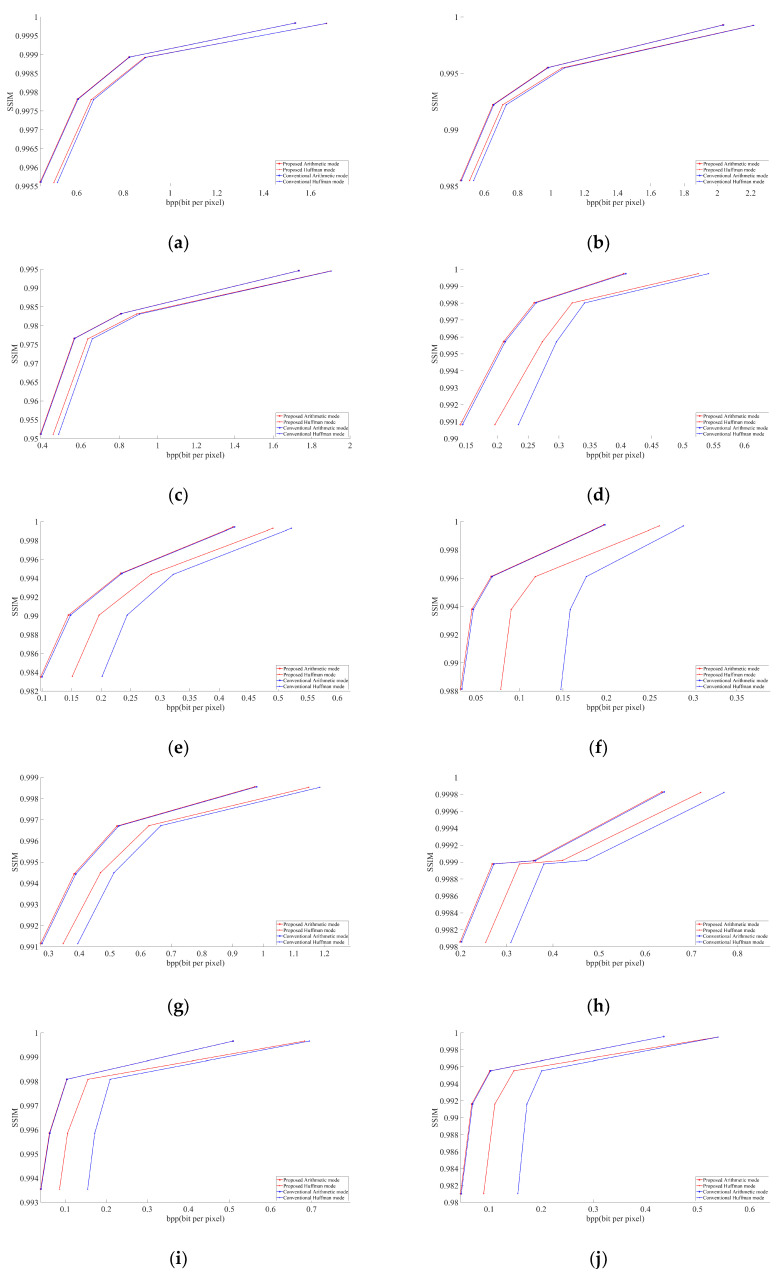

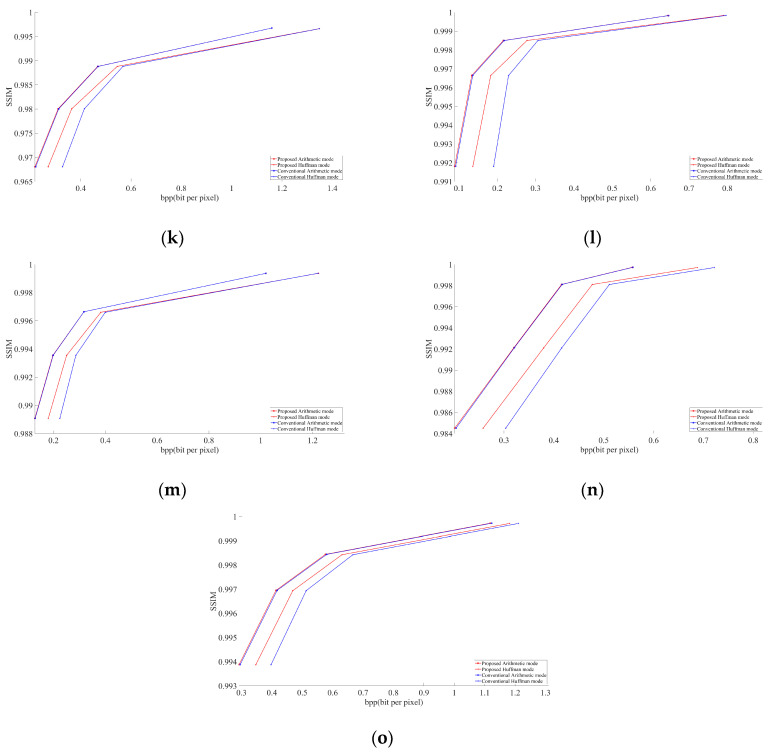
SSIM results of test images shown in Figure 4: (**a**) Street View, (**b**) Woman, (**c**) Night Scene (**d**) Jars, (**e**) Flowers, (**f**) Glasses, (**g**) Screenshot1, (**h**) Screenshot2, (**i**) Spoon, (**j**) Sunset, (**k**) Paris, (**l**) Ice Cream, (**m**) Air Jet, (**n**) Statue, (**o**) Icon.

We conducted an experiment on 15 test images using libjpeg-turbo [13] version 2.0.5. All test images were taken from the JPEG AI dataset [14]. These 15 images, shown in Figure 4, were selected carefully. They include two screenshots, two homogenous images, one image of night view, one image of street daytime view, one item close-up, one human close-up image, and seven additional random images. Thus, in these 15 test images, we included a broad variety of major types of images to obtain a useful and indicative result. Figure 5 and Figure 6 describe the graphical results for all the test images shown in Figure 4. The *Y*-axis represents the PSNR and SSIM values respectively in Figure 5 and Figure 6, whereas the *X*-axis represents the BPP.

As discussed in Section 2.1, chroma components Cb and Cr are downsampled to the 4:2:2 or 4:2:0 type in JPEG [4]. In this paper, we targeted the 4:2:0 subsampling at different QF ranging from low to high to obtain a better and clearer result. The selected QF values were 30, 50, 70, and 90. Graphs were obtained using MATLAB R2020a. We considered PSNR (peak signal-to-noise) and BPP (bits per pixel) to evaluate our obtained results. Compressed buffers after the step detailed in Section 3.2 for arithmetic and after the step is given in Section 3.3 for Huffman were included in the file size for calculating BPP. All the images decoded with the modified JPEG decoder had the same PSNR as the images decoded by the conventional JPEG decoder. This shows the successful implementation of our modified decoder.

In all images, our proposed approach achieved significant improvements. The demonstrated improvement in the case of homogeneous images was greater than for complex images. Among the test images used in the experiment, Figure 4f showed the best result. The proposed algorithm is tested only for high-resolution and original images. The lowest image resolution was 1980 × 1272 among the test images shown in Figure 5. Due to the high possibility of consecutive empty DCT 8 × 8 blocks, the proposed algorithm is considered useful in high-resolution images. To calculate the average gain in BPP for both Huffman and arithmetic mode, shown in Table 1, we used Bjontegaard’s metric [15,16,17].

In Table 2 and Table 3, for Huffman and Arithmetic encoding mode, respectively, we describe the test images actual file size encoded by the conventional JPEG encoder [13], file size when we filtered out the empty blocks, and the difference between actual file size and when we excluded the empty blocks from encoding. This difference indicates the size taken by the empty blocks in encoded images. We added another column of the proposed method. It shows the additional data required by the proposed method to encode the empty blocks and their locations. Moreover, the column named “Gain” in Table 2 and Table 3 represents the ratio of the size required to encode the empty blocks by the conventional JPEG encoder to the proposed JPEG encoder. All the sizes in Table 2 and Table 3 are calculated in bytes.

## 5. Conclusions

In this paper, we have proposed an improved version of the conventional JPEG algorithm. Based on experimental results, we concluded that the proposed algorithm increases the compression ratio. In other words, a higher quality image can be obtained at the same BPP.

A good balance between quality and BPP is always a major concern in the field of image processing. In the market, almost all social networks use JPEG encoders and compress images prior to sending. Compression is required due to storage resource restrictions on local and remote servers. If all users were to send and store images at their original quality, then storage space resource requirements would become an even more significant issue.

The conventional JPEG encoder uses both Huffman and arithmetic entropy encoding modes. Thus, to maintain an equivalent complexity level between the conventional and our proposed entropy coding, if the arithmetic encoding mode is selected in conventional JPEG, we used an arithmetic encoder to compress the additional data; otherwise, the Huffman encoder was used. Other than Huffman encoding and arithmetic encoding, the remaining steps are very simple. Thus, the complexity difference is negligible.

Our experimental results demonstrate that at the same PSNR value as the conventional JPEG encoder, the modified JPEG encoder shows better performance in terms of BPP, as shown in Figure 5 and Table 1. In the future, we may implement the same scenario in other image encoding methods.

## Figures and Tables

**Table 1:** Calculated Bjontegraad’s metric for BPP.

Images	BPP Gain	
	Huffman	Arithmetic
Street View	−0.6182%	−0.2383%
Woman	−0.7618%	−0.2160%
Night Scene	−2.0211%	−0.4155%
Jars	−7.7361%	−1.2956%
Flowers	−11.1837%	−0.3836%
Glasses	−28.8517%	−1.4458%
Screenshot1	−5.9439%	−0.9495%
Screenshot2	−11.0584%	−1.0505%
Spoon	−25.2422%	−0.7571%
Sunset	−23.903%	−1.0889%
Paris	−2.0695%	−0.3522%
Ice Cream	−10.1344%	−1.0378%
Air Jet	−4.0339%	−0.0535%
Statue	−7.1532%	−0.2405%
Icon	−5.6486%	−0.7301%

**Table 2:** Encoded images file size information for Huffman encoding mode.

Images	QF	Original Encoded File Size	Excluding Empty Blocks File Size	Difference	Proposed Method Additional Data Size	Gain
Street View	30	652,351	615,776	36,575	17,498	2.090239
	50	846,993	817,950	29,043	16,467	1.763709
	70	1,128,045	1,107,694	20,351	14,813	1.373861
	90	2,099,492	2,092,765	6727	9511	0.707286
Woman	30	329,175	306,748	22,427	7399	3.031085
	50	449,382	429,713	19,669	6591	2.984221
	70	663,933	648,092	15,841	6877	2.303475
	90	1,362,884	1,355,040	7844	5076	1.545311
Night Scene	30	195,362	178,543	16,819	5435	3.094572
	50	265,854	251,525	14,329	5096	2.811813
	70	365,488	353,724	11,764	4814	2.443706
	90	767,164	760,531	6633	4609	1.439141
Jars	30	356,342	280,478	75,864	18,054	4.20206
	50	449,985	394,850	55,135	20,420	2.700049
	70	519,012	469,248	49,764	19,361	2.570322
	90	823,986	778,846	45,140	19,835	2.275775
Flowers	30	61,583	43,227	18,356	2935	6.254174
	50	74,527	57,334	17,193	2683	6.408125
	70	98,380	83,120	15,260	3790	4.026385
	90	159,443	145,509	13,934	4378	3.182732
Glasses	30	45,330	22,687	22,643	1401	16.16203
	50	48,643	26,273	22,370	1507	14.84406
	70	54,325	33,165	21,160	3134	6.751755
	90	88,663	73,472	15,191	6662	2.280246
Screenshot1	30	226,949	196,064	30,885	3528	8.754252
	50	294,786	266,043	28,743	3578	8.033259
	70	382,787	356,753	26,034	3716	7.00592
	90	680,761	656,794	23,967	3232	7.415532
Screenshot2	30	239,702	191,024	48,678	6270	7.763636
	50	295,064	248,080	46,984	5914	7.944538
	70	367,033	321,095	45,938	5440	8.444485
	90	597,895	554,078	43,817	4646	9.431124
Spoon	30	346,814	185,789	161,025	7512	21.4357
	50	385,430	228,140	157,290	8816	17.84142
	70	469,380	329,066	140,314	19,385	7.238277
	90	1,555,706	1,505,563	50,143	23,760	2.110396
Sunset	30	312,033	169,844	142,189	10,366	13.71686
	50	346,847	211,625	135,222	11,532	11.72581
	70	404,130	279,721	124,409	16,993	7.321191
	90	1,084,243	1,048,243	36,000	34,870	1.032406
Paris	30	436,524	354,182	82,342	6047	13.617
	50	552,245	474,902	77,343	10,341	7.479257
	70	757,204	705,396	51,808	21,228	2.44055
	90	1,798,698	1,786,450	12,248	9927	1.233807
Ice Cream	30	533,325	360,830	172,495	20,457	8.432077
	50	642,160	487,456	154,704	24,491	6.316769
	70	857,003	740,336	116,667	36,672	3.181365
	90	2,230,815	2,189,367	41,448	25,609	1.618493
Air Jet	30	141,347	106,033	35,314	7200	4.904722
	50	180,196	149,887	30,309	7892	3.840471
	70	251,811	230,912	20,899	10,161	2.056786
	90	769,441	765,448	3993	5363	0.744546
Statue	30	227,527	188,009	39,518	5507	7.175958
	50	311,663	276,427	35,236	8015	4.396257
	70	383,561	349,665	33,896	8227	4.120092
	90	541,424	507,625	33,799	8207	4.118314
Icon	30	182,986	155,532	27,454	4364	6.291017
	50	236,248	210,801	25,447	5169	4.923003
	70	307,050	284,759	22,291	5790	3.849914
	90	558,022	539,548	18,474	5118	3.609613

**Table 3:** Encoded images file size information for arithmetic encoding mode.

Images	QF	Original Encoded File Size	Excluding Empty Blocks File Size	Difference	Proposed Method Additional Data Size	Gain
Street View	30	563,266	543,299	19,967	16,393	1.21802
	50	763,834	745,207	18,627	14,969	1.244372
	70	1,040,824	1,025,306	15,518	12,733	1.218723
	90	1,933,744	1,924,674	9070	7675	1.181759
Woman	30	284,075	274,875	9200	6197	1.484589
	50	403,134	394,583	8551	5456	1.567265
	70	604,432	596,354	8078	5421	1.490131
	90	1,250,988	1,244,910	6078	3931	1.546171
Night Scene	30	158,515	153,073	5442	4233	1.285613
	50	229,057	224,093	4964	3864	1.284679
	70	326,759	321,955	4804	3531	1.360521
	90	699,629	695,412	4217	3321	1.269798
Jars	30	219,022	198,674	20,348	15,047	1.352296
	50	324,088	302,158	21,930	17,739	1.236259
	70	400,090	378,217	21,873	17,421	1.255554
	90	620,514	597,811	22,703	17,940	1.265496
Flowers	30	30,529	27,844	2685	1954	1.374104
	50	45,240	42,427	2813	1715	1.640233
	70	71,772	68,150	3622	3008	1.204122
	90	130,042	126,119	3923	3173	1.236369
Glasses	30	10,348	9045	1303	857	1.52042
	50	14,417	12,976	1441	940	1.532979
	70	21,065	18,930	2135	1763	1.211004
	90	60,880	55,118	5762	5421	1.062904
Screenshot1	30	160,141	154,970	5171	2486	2.080048
	50	223,427	217,609	5818	2672	2.177395
	70	303,600	297,600	6000	2936	2.043597
	90	562,296	556,174	6122	2616	2.340214
Screenshot2	30	156,651	149,460	7191	4979	1.444266
	50	211,095	203,824	7271	4675	1.555294
	70	280,709	273,525	7184	4176	1.720307
	90	497,525	490,238	7287	3554	2.050366
Spoon	30	92,496	85,766	6730	4679	1.438342
	50	140,408	132,610	7798	5675	1.374097
	70	233,964	216,667	17,297	15,780	1.096134
	90	1,140,213	1,118,546	21,667	20516	1.056103
Sunset	30	94,009	82,482	11,527	8617	1.337705
	50	137,292	124,473	12,819	9588	1.336984
	70	206,631	191,088	15,543	12,932	1.201902
	90	874,864	842,381	32,483	32,252	1.007162
Paris	30	294,264	285,669	8595	4483	1.917243
	50	416,339	405,288	11,051	8001	1.381202
	70	625,035	604,641	20,394	18,344	1.111753
	90	1,546,620	1,539,256	7364	7196	1.023346
Ice Cream	30	255,679	229,832	25,847	18,736	1.379537
	50	380,400	350,686	29,714	22,358	1.32901
	70	609,735	570,596	39,139	32,946	1.187974
	90	1,810,811	1,785,977	24,834	21,223	1.170146
Air Jet	30	81,376	74,943	6433	5715	1.125634
	50	125,116	118,442	6674	6018	1.109006
	70	199,974	191,418	8556	8409	1.017481
	90	642,349	638,514	3835	3978	0.964052
Statue	30	152,776	146,856	5920	3861	1.533282
	50	240,670	233,264	7406	6700	1.105373
	70	312,434	305,055	7379	6545	1.127426
	90	419,035	411,561	7474	6520	1.146319
Icon	30	136,275	131,194	5081	3421	1.485238
	50	192,159	186,262	5897	4094	1.440401
	70	267,356	260,925	6431	4484	1.434211
	90	517,600	511,519	6081	3752	1.620736

## Data Availability

Restrictions apply to the availability of these data. All test images were obtained from JPEG-AI dataset. They are available at “JPEG AI image coding common test conditions”, ISO/IEC JTC1/SC29/WG1 N84035, 84th Meeting, Brussels, Belgium (July 2019).
